# A breastfeeding woman with spontaneous coronary artery dissection and possible takotsubo syndrome: A case report

**DOI:** 10.1097/MD.0000000000025775

**Published:** 2021-05-07

**Authors:** Makoto Takeuchi, Takenori Okada, Yuki Ikegami, Yumiko Nakamoto, Naomi Idei, Norihiko Ohashi

**Affiliations:** Department of Cardiology, Hiroshima Red Cross Hospital and Atomic-bomb Survivors Hospital, Hiroshima, Japan.

**Keywords:** breastfeeding, case report, recurrence, spontaneous coronary artery dissection, takotsubo syndrome

## Abstract

**Rationale:**

The relationship between spontaneous coronary artery dissection (SCAD) and takotsubo syndrome (TTS) remains unclear. Coexistence of SCAD and TTS has been reported in the literature. However, the relationship between these two diseases has not yet been elucidated.

**Patient concerns:**

A 36-year-old breastfeeding woman was brought to our hospital 52 days after cesarean section because of discomfort in her left arm and convulsions.

**Diagnoses:**

She was diagnosed of acute myocardial infarction (AMI). The convulsions were attributed to lethal arrhythmia.

**Interventions:**

An immediate coronary angiography revealed that her left anterior descending artery (LAD) was Type 2a SCAD, but with no flow limitation. In addition, a 12-lead electrocardiogram (ECG) revealed improvement in ST-elevation. We chose the conservative treatment according to the patient's needs.

**Outcomes:**

Conservative treatment was unsuccessful. She developed another acute myocardial infarction requiring another percutaneous coronary intervention (PCI) during hospitalization. From the course of hospitalization, we suspected the coexistence of SCAD and TTS.

**Lessons:**

When we treat patients with SCAD, we should consider the possibility of coexistence of TTS and confirm left ventricular wall motion. Patients with SCAD may require invasive treatment, hence, should be monitored for a while. An urgent strategy for managing patients with SCAD who require PCI should be established.

## 1 Introduction

Spontaneous coronary artery dissection (SCAD) and takotsubo syndrome (TTS) are 2 cardiovascular syndromes that occur predominantly in women.^[1]^ SCAD is a rare condition with an incidence of approximately 0.31% in patients with acute myocardial infarction (AMI).^[2]^ However, among women with AMI aged <50 years, SCAD is observed in approximately 35% of the cases.^[2]^ The Canadian SCAD study reported that 4.7% of women presenting with SCAD were in their peripartum period, and 2.9% were breastfeeding.^[3]^ Although involvement of female sex hormones and stress has been reported, the pathophysiology of SCAD remains unknown.

TTS was first described in 1991 and was thought to be a result of multivessel coronary artery spasms in absence of epicardial coronary artery disease.^[4]^ Approximately 1.7% of patients with suspected acute coronary syndrome have TTS. The average age of TTS onset is usually 65 years, with 15% of cases occurring in men and 85% in women.^[5]^

One of the diagnostic criteria for TTS is the absence of obstructive coronary disease.^[6]^ In addition, coexistence of coronary artery disease and TTS is unusual at presentation and challenging to manage successfully. Nevertheless, we encountered a case that could not be otherwise explained without considering the coexistence of SCAD and TTS. While reports of this coexistence of SCAD and TTS exist,^[7,8]^ they are quite rare, and the relationship between these 2 diseases has not yet been clarified.

Herein, we describe a rare case of a breastfeeding woman who had AMI associated with both left anterior descending artery (LAD) SCAD and apical ballooning-type TTS. We treated this rare condition conservatively. However, the patient developed another AMI during her hospitalization that necessitated advanced treatment to stabilize her condition.

## 2 Case report

A 36-year-old woman (gravida 1 and para 1) gave birth to a boy by cesarean section approximately 2 months prior to her presentation. She had hyperlipidemia that represented a risk factor for coronary heart diseases and a history of migraines. She was unfamiliar with parenting, as this was her first baby; nevertheless, she had not recently experienced any remarkable mental or physical stress.

Approximately 51 days after the cesarean section, she noticed discomfort in her left arm while breastfeeding in the evening. However, this discomfort had improved in a few minutes. On the following night, she made an emergency visit to our hospital because she experienced loss of consciousness with convulsions that lasted for 10 seconds during breastfeeding. She felt discomfort in her left arm for 20 minutes after the convulsions until she was brought to our hospital.

**Figure 1:**
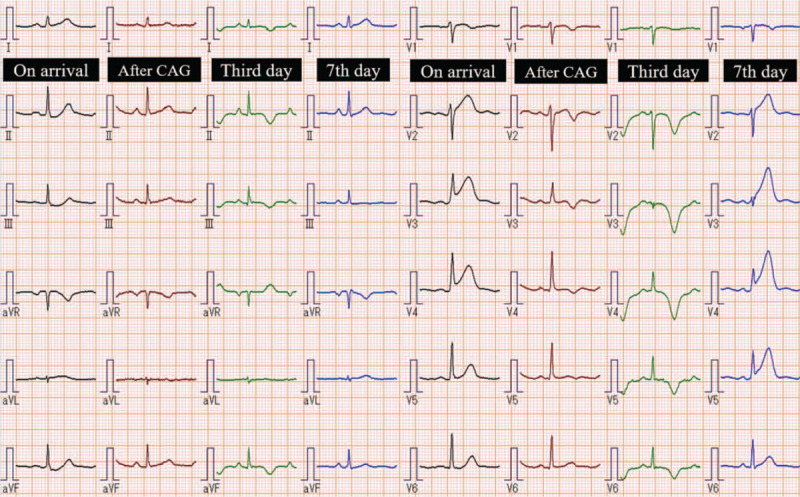
12-lead electrocardiogram during hospitalization.

**Figure 2:**
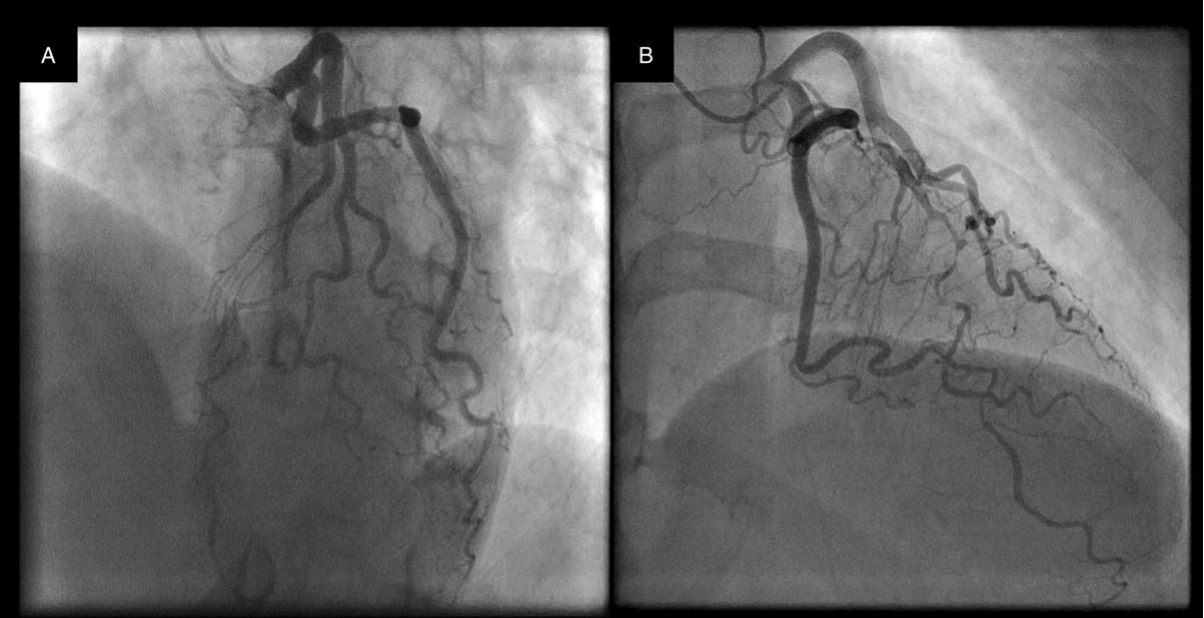
Coronary angiography on the first day of hospitalization. (A) We observed spontaneous coronary artery dissection in the left anterior descending artery with no flow limitations. (B) The patient had a non-wrapped left anterior descending artery.

On admission, she had normal physical examination findings, with blood pressure of 152/84 mm Hg, heart rate of 82/min, and respiratory rate of 14/min. She had a feeling of discomfort in her left arm during her visit. Accordingly, 12-lead electrocardiogram (ECG) revealed ST elevation in the precordial leads with reciprocal changes in the inferior leads (Fig. 1). Transthoracic echocardiography revealed extensive akinesis centered at the apex of the left ventricle. Thus, we suspected acute coronary syndrome and performed coronary angiography (CAG) immediately (first day of hospitalization), which revealed type 2a SCAD in the middle-to-distal LAD (Fig. 2a).

Her convulsions were thought to be due to lethal arrhythmias and reduced cerebral perfusion subsequent to AMI. However, the LAD presented with no flow limitations (thrombolysis in myocardial infarction [TIMI] grade 3). A repeat 12-lead ECG revealed improvement in ST elevation. Conservative treatment was initiated with bisoprolol (2.5 mg), because she wanted to breastfeed and desired to have a second child. Her baseline creatine phosphokinase was approximately 80 IU/L. However, its level rose to 286 IU/L during this event. Her troponin I levels increased to 6.30 ng/mL, suggesting that the area of myocardial infarction was not extensive.

**Figure 3:**
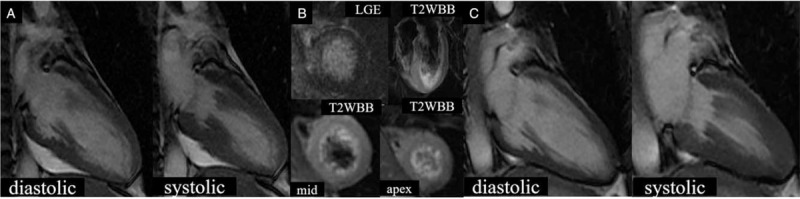
(A) Apical ballooning observed on cardiovascular magnetic resonance imaging (CMR) on the fourth day of hospitalization. (B) Late gadolinium enhancement and T2-weighted black blood are observed on CMR on day 4 of hospitalization. (C) Second CMR 49 days after the first CMR.

However, a 12-lead ECG performed on the third day of hospitalization revealed ECG changes suggestive of TTS (Fig. 1). On the fourth day of hospitalization, cardiovascular magnetic resonance imaging (CMR) detected apical ballooning. This finding was considered inconsistent with the perfusion area of the LAD, thus increasing the likelihood of TTS and SCAD coexistence (Fig. 3a). We observed late gadolinium enhancement only in the apex of the anterior wall (Fig. 3b), which coincided with the perfusion area of the LAD. We also observed a high signal with T2-weighted black blood consistent with the apical ballooning region (Fig. 3b).

**Figure 4:**
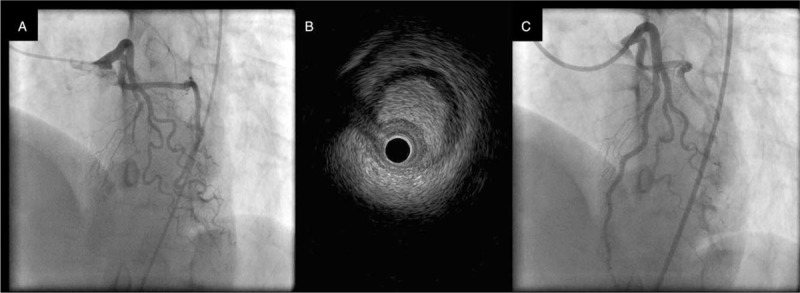
(A) Coronary angiography on the seventh day of hospitalization. The left anterior descending artery flow was TIMI grade 0. (B) We performed intravascular ultrasonography after balloon dilatation of the lesion. The true lumen was occluded by an intramural hematoma that developed in the false lumen. (C) We performed percutaneous coronary intervention, and the left anterior descending artery became TIMI grade 3.

**Figure 5:**
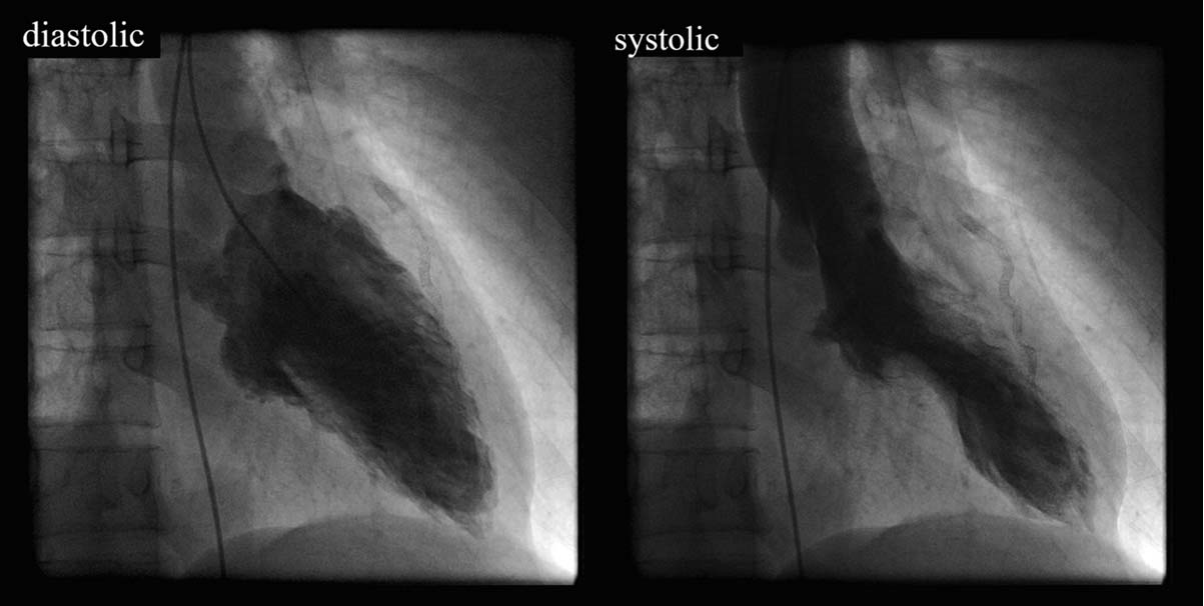
Left ventriculography on the seventh day of hospitalization.

Subsequently, the patient's condition stabilized. However, in the morning of the seventh day of hospitalization, she felt chest discomfort while returning from the toilet to her hospital room. A 12-lead ECG revealed ST elevation in the precordial lead (Fig. 1), and a second CAG was then performed. Since the LAD showed TIMI grade 0, we decided to perform percutaneous coronary intervention (PCI) (Fig. 4a). Intravascular ultrasonography was performed after balloon dilatation of the lesion. However, the true lumen was occluded by an intramural hematoma that developed in the false lumen (Fig. 4b). Finally, 2 everolimus-eluting stents were placed from the middle to the distal LAD and were able to achieve adequate revascularization (Fig. 4c). Since we suspected the existence of TTS from the beginning, we performed left ventriculography (Fig. 5). Similar to the CMR findings, left ventriculography revealed apical ballooning and hyperkinesis in the basal segment, which did not coincide with the perfusion area of the LAD. Therefore, we concluded that SCAD and TTS might be coexisting. After the second myocardial infarction, creatine phosphokinase and troponin I levels increased to 106 IU/L and 1.02 ng/mL, respectively.

We detected no evidence of connective tissue disease. The patient received dual antiplatelet therapy with aspirin 100 mg and prasugrel 3.75 mg for maintenance and rosuvastatin 5 mg and bisoprolol 5 mg for secondary prevention. The treatment significantly improved her left ventricular wall movement, and she was discharged to home 18 days after admission. The second CMR performed 49 days after the first revealed an improvement in wall motion abnormalities and recovery of the left ventricular ejection fraction from 39% to 60% (Fig. 3c).

This study was approved by the institutional review board of the Hiroshima Red Cross Hospital and Atomic-bomb Survivors Hospital. Informed written consent was obtained from the patient for the publication of this case report and associated images.

## 3 Discussion

Although cases of SCAD and TTS coexistence have been reported in the literature,^[8]^ such coexistence is quite rare. Moreover, the relationship between these 2 diseases has not yet been elucidated. One of the diagnostic criteria for TTS includes the absence of obstructive coronary disease.^[6]^ However, recently, there has been a move to update its criteria from the International Takotsubo Diagnostic Criteria.^[9]^ These criteria make it possible to diagnose TTS in patients with pre-existing SCAD. A retrospective review of CAG in patients diagnosed with TTS reported that 2.5% of the cases met the criteria for SCAD diagnosis.^[10]^ Given the many similarities in patients’ demographics, clinical presentation, and predisposing stressors between SCAD and TTS,^[11]^ co-occurrence of the 2 diseases is not accidental, and they are considered related to each other. There continues to be much chicken-or-egg debate regarding the overlap of SCAD and TTS.^[11]^ Y-Hassan et al reported a 54-year-old woman who presented with an obtuse marginal SCAD and TTS.^[7]^ Ghafoor et al reported a 52-year-old woman who presented with right coronary artery SCAD and TTS.^[8]^

We first suspected the existence of TTS because it caused changes in the 12-lead ECG associated with apical ballooning. We observed no abnormal Q-waves on her 12-lead ECG. A 12-lead ECG on the third day of hospitalization also revealed negative T-waves in 9 leads (Fig. 1). Nevertheless, we suspected that TTS and SCAD coexisted because of positive T-waves in lead aVR and the lack of negative T-waves in lead V1. Kosuge et al reported that positive T-waves in lead aVR and absence of negative T-waves in lead V1 identified TTS with 94% sensitivity and 95% specificity, which represent the highest diagnostic accuracy.^[12]^ The maximum amplitude of negative T-waves revealed a high value of 1.0 mV, and the maximal QTc interval revealed an extension of 640 ms (Bazett's formula). In this patient, the 12-lead ECG changes did not coincide with the infracted area of the anterior wall, which were typical ECG changes due to TTS.

Eitel et al reported myocardial edema in 81% of patients with TTS on CMR^[13]^ and that CMR may help establish a diagnosis of TTS. However, repeat CMR performed within 6 months revealed disappearance of the myocardial edema, which was similar to our observation in this case. We observed a high signal with T2-weighted black blood consistent with the apical ballooning region (Fig. 3b). These results suggested that myocardial edema was present around the left ventricle. We suspected the coexistence of TTS because it did not match the perfusion area of the LAD. The second CMR performed 49 days after the first demonstrated disappearance of myocardial edema.

It may be somewhat difficult to compare CAG and CMR. However, we could perform left ventriculography because the patient developed a second AMI on the seventh day of hospitalization (Fig. 5). CAG revealed that the LAD was non-wrapped (Fig. 2b), and left ventriculography revealed mid-apical wall motion abnormality of the left ventricle (Fig. 5). Left ventricular angiography might not show the typical apical ballooning pattern of TTS. However, a long time had elapsed since the onset of TTS, and apical ballooning might have been weakened. Its occurrence might be also caused by β-blockers that attenuated the wall motion abnormality. As a result, we suspected that SCAD and TTS might coexist.

We suggested that her convulsions might have been caused by a reduction in cerebral perfusion due to lethal arrhythmias. She had frequent and repeated ventricular extrasystoles, which disappeared with the administration of β-blocker. This frequent arrhythmia suggests that a lethal arrhythmia might have occurred. However, convulsions may be caused by epilepsy. In addition, head magnetic resonance imaging and electroencephalography should be considered if convulsions are repeated. Medina et al reported that TTS was involved in the central autonomic nervous system.^[14]^ If convulsions recur, an association between epilepsy and TTS should be more importantly taken into account in the differential diagnosis than the relationship between SCAD and TTS.

In the Canadian SCAD study, 4% of patients with SCAD had in-hospital recurrent myocardial infarction and 2.5% underwent unplanned revascularization.^[3]^ Despite her stable symptoms, our patient developed AMI on the seventh day of hospitalization.

We did not perform PCI when the first CAG was TIMI grade 3, as the procedural failure rate of SCAD has been reported to reach as high as 53%.^[15]^ Because the second CAG on the seventh day of hospitalization was TIMI grade 0, we decided to perform PCI. Thus, there is a need for urgently establishing a strategy for managing patients with SCAD who require PCI.

## Author contributions

**Conceptualization:** Makoto Takeuchi.

**Writing – original draft:** Makoto Takeuchi.

**Writing – review & editing:** Takenori Okada, Yuki Ikegami, Yumiko Nakamoto, Naomi Idei, Norihiko Ohashi.
